# Dosimetric impact of port number in TomoDirect‐based lower‐body total body irradiation

**DOI:** 10.1002/acm2.70504

**Published:** 2026-02-16

**Authors:** Yoshiki Suetsugu, Naoki Higashi, Yukihide Fukuyama, Yuka Yamanaka, Keiki Inoue, Shun Nakano, Hiromi Terashima, Tomonari Sasaki

**Affiliations:** ^1^ Department of Radiology Hospital of University of Occupational and Environmental Health Kitakyushu City Fukuoka Japan; ^2^ Department of Radiology Harasanshin Hospital Fukuoka City Fukuoka Japan; ^3^ Department of Radiology Fukuoka Tokushukai Hospital Kasuga City Fukuoka Japan; ^4^ Department of Radiology Hamano‐machi Hospital Fukuoka City Fukuoka Japan; ^5^ Department of Radiology Steel Memorial Yawata Hospital Kitakyushu City Fukuoka Japan; ^6^ Department of Radiology Saga‐ken Medical Centre Koseikan Saga City Saga Japan; ^7^ Department of Radiation Oncology Iizuka Hospital Iizuka City Fukuoka Japan

**Keywords:** TomoDirect, TomoHelical, TomoTherapy, total body irradiation

## Abstract

**Background:**

The feasibility of total body irradiation (TBI) delivery using the TomoDirect mode of a TomoTherapy system has been demonstrated. In clinical practice, TomoTherapy‐based TBI is typically performed in two parts: upper and lower body.

**Purpose:**

This study aimed to evaluate the dosimetric impact of varying port numbers on dose evaluation indices for lower body irradiation using TomoDirect and to compare these results with those of TomoHelical in a simulation setting.

**Methods:**

Sixteen patients who underwent myeloablative TBI using TomoHelical between October 2017 and March 2021 were retrospectively analyzed. TomoDirect plans with 2 to 12 ports at approximately equal beam angles were generated (modulation factor = 1.5, field width = 5.0 cm, pitch = 0.500). TomoHelical plans used identical parameters except for a pitch of 0.397. The prescribed dose was 12 Gy in six fractions. Dose indices (D2, D98, D50, homogeneity index [HI]) and beam‐on time were compared.

**Results:**

In TomoDirect plans, all dose evaluation indices worsened as the number of ports increased up to five, but changes became minimal beyond eight ports. D2 was significantly improved in all TomoDirect plans compared with those of TomoHelical plan. D98 was significantly lower for the three‐ and five‐port TomoDirect plans, and no TomoDirect plan achieved higher D98 than TomoHelical. The D50 and HI were significantly improved in all TomoDirect plans except the five‐port configuration. The two‐port TomoDirect plan achieved the most favorable dose indices and the shortest beam‐on time.

**Conclusions:**

The two‐port TomoDirect approach with anterior‐posterior beam configuration provides an efficient and clinically reasonable option for lower‐body irradiation, offering improved dose homogeneity and reduced treatment time compared with TomoHelical.

## 1 Introduction

Total body irradiation (TBI) is frequently used as a conditioning regimen for allogeneic hematopoietic stem cell or bone marrow transplantation. When combined with chemotherapy, TBI eradicates residual malignant cells and provides immunosuppression to reduce donor cell rejection.^1^, ^2^, ^3^

TomoTherapy (Accuray, Sunnyvale, CA, USA) delivers radiation using a continuously rotating fan beam in conjunction with couch translation. The conventional mode, TomoHelical, achieves optimal dose distribution and conformity through 360° gantry rotation.^4^, ^5^, ^6^ More recently, TomoDirect, a novel mode for TomoTherapy, was introduced, allowing delivery through 2–12 discrete angles with a fixed gantry as the couch moves longitudinally.^7^, ^8^, ^9^ Several studies have demonstrated the clinical feasibility and dosimetric advantages of TomoHelical‐based TBI compared with linear accelerator‐based approaches, including the extended source‐to‐surface distance (SSD) method.^10^, ^11^ In recent years, irradiation techniques using conventional C‐arm or other O‐ring linear accelerators, employing volumetric modulated arc therapy or anterior‐posterior (AP/PA) beam configurations at standard SSD, have been reported.^12^, ^13^, ^14^, ^15^, ^16^ All of these techniques require treatment to be divided into multiple segments due to limited field length. An increased number of junctions between segments makes treatment planning more time‐consuming. TomoTherapy‐based TBI is performed in two divided segments, with a junction typically positioned at the mid‐thigh. This approach is possible because TomoTherapy allows coverage of extended lengths in a single irradiation (up to 135 cm),^4^, ^5^ which minimizes the number of segments required and simplifies the planning process.

Recent investigations have explored TBI using TomoDirect. Salz et al. reported potential advantages of TomoDirect over TomoHelical, including lower mean dose rates (reducing interstitial pneumonia risk), improved dose uniformity in circulating blood cells, and a beam expansion function that mitigates setup errors.^7^ Kasai et al. examined the relationship between port number and dose indices in low‐dose upper‐body TBI, showing that TomoDirect plans with eight or more ports can significantly improve dose homogeneity compared with TomoHelical.^8^ Moreover, Broggi et al. described the clinical implementation of low‐dose TBI using TomoDirect.^9^

While previous studies employed two or four ports for lower‐body TBI with TomoDirect,^4^, ^7^, ^9^ the effect of port number on dosimetric indices for lower‐body TBI remains unclear. Owing to the pronounced geometric differences between the upper and lower body, including bilateral limb separation resulting in substantial air gaps between the limbs, it is plausible that port number selection in lower‐body TBI leads to dosimetric trends different from those observed in upper‐body treatments. This study addresses this gap by evaluating the impact of port number in TomoDirect‐based TBI, comparing results with those of TomoHelical, and providing a clinical rationale for selecting the optimal irradiation approach for the lower body in terms of target dose homogeneity and beam‐on time.

## 2 Materials and Methods

### 2.1 Patients

Sixteen patients received TBI via the TomoHelical mode of TomoTherapy with a prescribed dose level of 12 Gy delivered in six fractions over three days between October 2017 and March 2021. To study a homogeneous cohort, no patients with prescription doses different from 12 Gy were included. This study was a retrospective analysis, and all eligible consecutive patients treated during the study period were included without additional selection criteria. Patient details are presented in Table 1. The cohort consisted of 10 males and 6 females, with a median age of 35 (range: 20–55) years at the time of treatment, exhibiting significant physical variability with a body mass index (BMI) range of 13.2 to 23.6 kg/m^2^. This retrospective study protocol was reviewed and approved by the Institutional Review Board of Harasanshin Hospital (Approval No. 2016‐10).

**TABLE 1:** Patient characteristics.

Patient no.	Age (years)	Sex	Diagnosis	Height (cm)	Weight (kg)	BMI
1	29	F	ALL	156.5	48.8	19.9
2	20	M	AML	183	62.3	18.6
3	20	F	AML	162.4	34.8	13.2
4	29	M	ALL	177.4	50.8	16.1
5	49	M	ATLL	174	44.7	14.8
6	50	M	MDS	172.1	61.3	20.7
7	36	F	ALL	165	56.5	20.8
8	34	M	AML‐MRC	170	65.1	22.5
9	49	F	ALL	165.5	63.5	23.2
10	20	M	ALL	167	47.2	16.9
11	48	M	MF	181	67.5	20.6
12	20	M	AML	172.5	56.7	19.1
13	28	F	AML	159	50.5	20.0
14	55	M	AML‐MRC	168.3	43.3	15.3
15	45	M	AML	175	52.7	17.2
16	45	F	ALL	161.4	61.4	23.6

ALL: acute lymphoid leukemia, AML: acute myelogenous leukemia, AML‐MRC: acute myeloid leukemia with myelodysplasia‐related changes, ATLL: adult T‐cell leukemia/lymphoma, BMI: body mass index [= weight (kg)/ height^2^ (m)], F: female, M: male, MDS: myelodysplastic syndromes, MF: myelofibrosis.

### 2.2 Immobilization and acquisition of computed tomography (CT) images

The patient was immobilized as described by Kasai et al.^8^ Briefly, immobility was achieved using a Head and Neck Vac Lock Cushion, a thermoplastic head mask, and the Uni‐frame Patient Positioning System (CIVCO, IA, USA) for head and neck support. Body Support II, ESS‐15, and ESF‐19HN (Engineering System Co., Ltd., Nagano, Japan) were used for body and lower‐extremities support. A radio‐opaque marker was placed in the middle of the thigh at the junction plane.

Two planning CT images (SCENARIA, Hitachi, Tokyo, Japan) with a 5 mm slice thickness were acquired in the supine position: one for the upper body head‐first oriented (from the vertex to the middle of the thigh), and one for lower‐body feet‐first oriented (from the feet to the middle of the thigh). This approach was required because the longitudinal maximum couch travel of the TomoTherapy system is limited to 135 cm.^4^, ^5^ The CT image sets were transferred to a Pinnacle^3^ (Philips Medical Systems, Eindhoven, Netherlands) for structure delineation.

### 2.3 Structure delineation

As this study focused on lower‐body TBI, only CT image sets with a feet‐first orientation were used for structure delineation. The clinical target volume (CTV) was defined as the contour of the lower limb below the junction plane. Two planning target volumes (PTVs) were created: PTV1 and PTV2. PTV1 was obtained from the CTV, with an inner margin of 5 mm under the skin surface to avoid dose inhomogeneity near the skin surface. The PTV2 was obtained from the CTV with an air margin of 10 mm. We did not define organs at risk for the lower‐body plans in this study, because unlike upper‐body TBI, which necessitates the protection of critical structures such as the lungs and kidneys, the lower‐body region contains no such dose‐limiting organs. The lower planning CT and structural data were exported to the TomoTherapy planning station (TomoHDA ver. 5.1.0.4, Accuray, CA, USA).

### 2.4 Treatment planning and plan evaluation

Treatment planning was performed at the TomoTherapy planning station. TomoDirect and TomoHelical treatment plans were generated with a prescription dose of 12 Gy in six fractions prescribed to cover PTV1 with 85% isodose line.

For the TomoDirect plans, treatment plans using 2–12 ports with approximately equidistant beams were generated, as described by Kasai et al.^8^ The specific gantry angles (beam angles) for each port configuration are detailed in Table 2. For the two‐port configuration, two plans were generated using AP/PA and bilateral (LR/RL) beams. The TomoTherapy parameters—field width (FW), modulation factor (MF), and pitch—were set to 5 cm (dynamic jaw mode), 1.5, and 0.500, respectively. These values were determined based on previous studies of TomoDirect‐based TBI.^7^, ^8^, ^9^

**TABLE 2:** Beam angles in TomoDirect plans.

Number of ports	Beam angles (degrees)
2 (AP/PA)	0, 180
2 (LR/RL)	90, 270
3	0, 120 240
4	0, 90, 180, 270
5	0, 72, 144, 216, 288
6	0, 60, 120, 180, 240, 300
7	0, 51, 103, 154, 206, 257, 309
8	0, 45, 90, 135, 180, 225, 270, 315
9	0, 40, 80, 120, 160, 200, 240, 280, 320
10	0, 36, 72, 108, 144, 180, 216, 252, 288, 324
11	0, 32, 65, 98, 131, 164, 197, 230, 263, 296, 328
12	0, 30, 60, 90, 120, 150, 180, 210, 240, 270, 300, 330

For TomoHelical planning, FW and MF were set to the same values as those of the TomoDirect plans. Two TomoHelical plans with pitch values of 0.397 and 0.430 were generated, in the same manner as previously reported for upper‐body TBI.^8^ The normal dose grid was used during optimization (100 iterations), and the fine dose grid was applied for final dose calculation in both irradiation modes.

Dose‐volume histogram (DVH) parameters including the dose received by 2% of the PTV1 (D2), 98% of the PTV1 (D98), the median dose of the PTV1 (D50), and homogeneity index (HI), were evaluated. HI was defined by ICRU report 83 using the following equation: HI = (D2 ‐ D98)/D50.^17^ In addition, the beam‐on time was obtained for each plan. Following the American College of Radiology (ACR) and American Society for Radiation Oncology (ASTRO) practice guidelines, dose inhomogeneity was maintained within ± 10%.^2^ Accordingly, the criteria of D2 ≤ 13.2 Gy and D98 ≥ 10.8 Gy were established to assess whether the treatment plans satisfied these conditions.

### 2.5 Statistical analysis

The Wilcoxon signed‐rank test was used to compare treatment plans with different settings. Specifically, TomoHelical plans with pitch values of 0.397 and 0.430, and TomoDirect plans using AP/PA and LR/RL beam configurations, were compared. For both comparisons, statistical significance was set at 5%, and the superior plan was identified. Subsequently, the Wilcoxon signed‐rank test was applied to compare TomoHelical and TomoDirect treatment plans, as well as TomoDirect plans using two ports with those utilizing different number of ports.

Statistical significance was defined as *P* < 0.05/11 = 0.0045 and *P* < 0.05/10 = 0.005 using Bonferroni correction. All statistical analyses were performed using the JMP Pro 16 software (SAS Institute Inc., Cary, NC, USA).

## 3 Results

**FIGURE 1:**
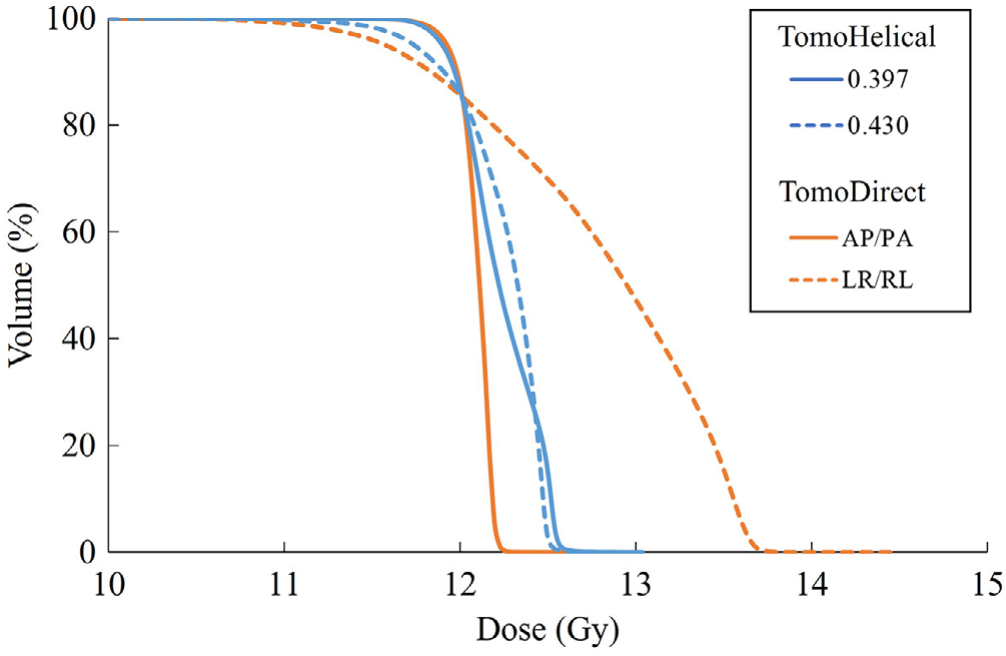
Dose volume histograms comparing TomoHelical plans with pitch values of 0.397 and 0.430, and TomoDirect plans using two‐ports with AP/PA and LR/RL beam configurations for patient 12, whose body mass index corresponds to the median of the cohort.

**FIGURE 2:**
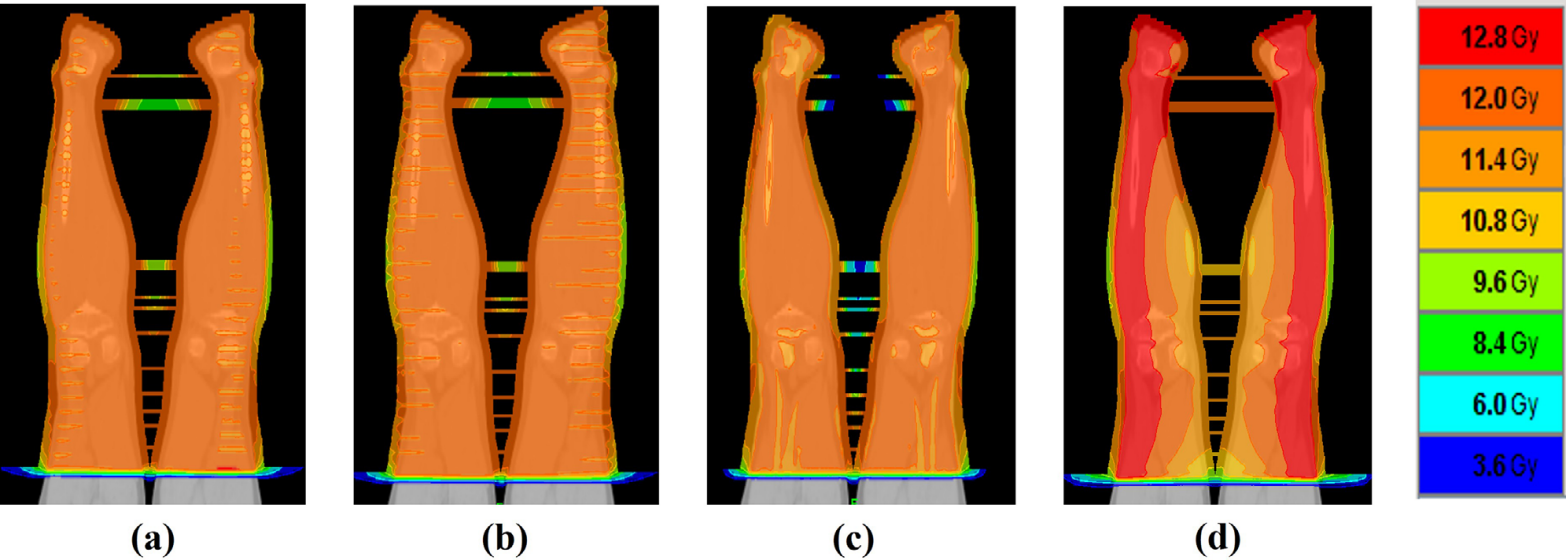
Dose distributions for patient 12, whose body mass index corresponds to the median of the cohort. (a) TomoHelical plan (pitch = 0.397), (b) TomoHelical plan (pitch = 0.430), (c) TomoDirect plan (two‐ports with AP/PA beam configuration), (d) TomoDirect plan (two‐ports with LR/RL beam configuration).

Table 3 summarizes the differences in pitch values for TomoHelical and beam configurations for the two‐port TomoDirect plans, while Figures 1 and 2 respectively show the DVH and dose distributions for the same patient whose BMI represents the median of the cohort.

**TABLE 3:** Differences in pitch value for TomoHelical and beam configuration for the two‐port TomoDirect (pitch = 0.500).

	TomoHelical			TomoDirect		
	0.397	0.430	*P*‐value	AP/PA	LR/RL	*P*‐value
D2 (Gy)	12.55 (12.45–12.57)	12.49 (12.39–12.58)	0.0194	12.21 (12.17–12.28)	13.42 (13.18–13.89)	< 0.0001
D98 (Gy)	11.82 (11.80–11.85)	11.61 (11.48–11.71)	< 0.0001	11.84 (11.75–11.89)	11.41 (11.00–11.57)	< 0.0001
D50 (Gy)	12.22 (12.19–12.25)	12.32 (12.24–12.38)	< 0.0001	12.11 (12.09–12.14)	12.81 (12.62–13.12)	< 0.0001
HI	0.058 (0.053–0.062)	0.073 (0.056–0.084)	< 0.0001	0.031 (0.023–0.040)	0.156 (0.134–0.242)	< 0.0001

D2: dose received by 2% of the volume; D50: median dose; D98: dose received by 98% of the volume; HI: homogeneity index [= (D2–D98)/ D50].

For TomoHelical, the D2 for the 0.397 pitch was significantly less favorable than that for the 0.430 pitch. In contrast, the D98, D50, and HI for the 0.397 pitch showed significant improvement over the 0.430 pitch. Although the 0.430 pitch produced a smaller high‐dose region, the 0.397 pitch achieved a more homogeneous dose distribution. Therefore, the data obtained from the 0.397 pitch were used for subsequent comparison between the TomoDirect and TomoHelical plans.

In comparing beam configurations for TomoDirect, all dose evaluation indices for the AP/PA beam configuration were significantly better than those for the LR/RL plan. The AP/PA configuration provided a more homogeneous dose distribution, whereas the LR/RL plan showed an increased high‐dose region, as evident in the DVH. The median value of D2 for the LR/RL configuration exceeded 13.2 Gy (+10% of the prescribed dose). Accordingly, data from the AP/PA configuration were used for comparisons among TomoDirect plans with different numbers of ports.

**FIGURE 3:**
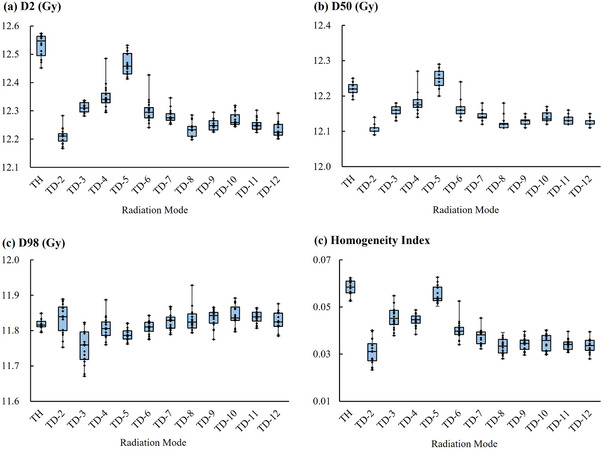
Dose evaluation indices for varying numbers of ports in the TomoDirect plans and for the TomoHelical plan (*n* = 16). (a) D2, dose received by 2% of the volume; (b) D98, dose received by 98% of the volume; (c) D50, median dose; (d) homogeneity index = (D2–D98)/D50. TD‐n: TomoDirect n‐port (*n* = 2–12), TH: TomoHelical with the 0.397 pitch.

Table 4 and Figure 3 present the dosimetric results for the TomoDirect plans with 2–12 ports and the TomoHelical plans. Some indices showed nominal significance at *P* < 0.05 but did not maintain statistical significance after the Bonferroni correction (*P* < 0.0045 and *P* < 0.005). Dose evaluation indices tended to worsen with increasing port number up to five, with minimal changes beyond eight ports. While some statistical variations were observed beyond eight ports, the absolute differences were minimal and considered clinically negligible. The D2 of all TomoDirect plans was significantly improved compared with that of the TomoHelical plan. Conversely, the D98 values of TomoDirect plans with three and five ports were significantly worse than those of TomoHelical plan, and no TomoDirect plan achieved a significantly better D98 than TomoHelical. The D50 and HI of all TomoDirect plans, except the five‐port plan, were significantly improved compared to those of TomoHelical.

**TABLE 4:** Dose evaluation indices and statistical analysis results.

Radiation mode	D2 (Gy)	*P*‐value (v.s. TH)	*P*‐value (v.s. TD‐2)	D98 (Gy)	*P*‐value (v.s. TH)	*P*‐value (v.s. TD‐2)	D50 (Gy)	*P*‐value (v.s. TH)	*P*‐value (v.s. TD‐2)	HI	*P*‐value (v.s. TH)	*P*‐value (v.s. TD‐2)
TD‐2	12.21 (12.17–12.28)	<0.0001^*^	–	11.84 (11.75–11.89)	0.1362	–	12.11 (12.09–12.14)	<0.0001^*^	–	0.031 (0.023–0.040)	<0.0001^*^	–
TD‐3	12.31 (12.28–12.34)	<0.0001^*^	<0.0001^†^	11.76 (11.67–11.82)	0.0003^*^	0.0002^†^	12.16 (12.13–12.18)	<0.0001^*^	<0.0001^†^	0.045 (0.038–0.055)	<0.0001^*^	<0.0001^†^
TD‐4	12.34 (12.30–12.49)	<0.0001^*^	<0.0001^†^	11.81 (11.76–11.89)	0.0827	0.0457	12.18 (12.14–12.27)	0.0001^*^	<0.0001^†^	0.045 (0.038–0.049)	<0.0001^*^	<0.0001^†^
TD‐5	12.46 (12.41–12.53)	0.0003^*^	<0.0001^†^	11.79 (11.76–11.82)	0.0001^*^	0.0018^†^	12.25 (12.20–12.29)	0.0018	<0.0001^†^	0.054 (0.050–0.063)	0.0226	<0.0001^†^
TD‐6	12.30 (12.24–12.43)	<0.0001^*^	<0.0001^†^	11.81 (11.78–11.84)	0.2655	0.0522	12.16 (12.13–12.24)	<0.0001^*^	<0.0001^†^	0.040 (0.034–0.053)	<0.0001^*^	<0.0001^†^
TD‐7	12.28 (12.25–12.35)	<0.0001^*^	<0.0001^†^	11.83 (11.79–11.87)	0.5214	0.3654	12.14 (12.12–12.18)	<0.0001^*^	<0.0001^†^	0.038 (0.032–0.045)	<0.0001^*^	0.0014^†^
TD‐8	12.23 (12.20–12.29)	<0.0001^*^	0.0128	11.82 (11.79–11.93)	0.4504	0.5092	12.12 (12.11–12.18)	<0.0001^*^	0.0068	0.033 (0.028–0.039)	<0.0001^*^	0.1468
TD‐9	12.25 (12.23–12.30)	<0.0001^*^	0.0001^†^	11.84 (11.78–11.87)	0.0363	0.7062	12.13 (12.11–12.15)	<0.0001^*^	0.0006^†^	0.035 (0.030–0.040)	<0.0001^*^	0.0734
TD‐10	12.26 (12.24–12.32)	<0.0001^*^	<0.0001^†^	11.84 (11.80–11.89)	0.0135	0.8801	12.14 (12.12–12.17)	<0.0001^*^	<0.0001^†^	0.036 (0.030–0.040)	<0.0001^*^	0.0302
TD‐11	12.25 (12.22–12.30)	<0.0001^*^	0.0002^†^	11.84 (11.81–11.86)	0.0049	0.8652	12.13 (12.12–12.16)	<0.0001^*^	0.0002^†^	0.034 (0.031–0.040)	<0.0001^*^	0.0621
TD‐12	12.22 (12.20–12.29)	<0.0001^*^	0.0184	11.82 (11.79–11.88)	0.3261	0.2911	12.13 (12.11–12.15)	<0.0001^*^	0.001^†^	0.034 (0.028–0.040)	<0.0001^*^	0.1467
TH	12.55 (12.45–12.57)	<0.0001^*^	–	11.82 (11.80–11.85)	–	–	12.22 (12.19–12.25)	–	–	0.058 (0.053–0.062)	<0.0001^*^	–

TD‐n: TomoDirect n‐port (*n* = 2–12), TH: TomoHelical (pitch = 0.397).

D2: the dose received by 2% of the volume, D50: median dose, D98: the dose received by 98% of the volume, HI: homogeneity index [= (D2‐D98)/ D50].

^*^ *P* < 0.0045, ^†^ *P* < 0.005.

The two‐port TomoDirect plan demonstrated the most favorable results across all dose evaluation indices. However, the differences between the two‐port plan and plans with eight or more ports were not statistically significant.

Table 5 shows the beam‐on time for TomoDirect plans with 2–12 ports and for the TomoHelical plans. The two‐port TomoDirect plan (AP/PA configuration) achieved the shortest beam‐on time. All TomoDirect plans required less beam‐on time than the TomoHelical plan with a 0.397 pitch, although beam‐on time tended to increase for TomoDirect plans with six or more ports.

**TABLE 5:** Beam‐on times of the TomoDirect and TomoHelical plans (mean ± SD).

Radiation mode	Beam‐on time (sec)
TD‐2	566 ± 56.8
TD‐3	549 ± 47.4
TD‐4	595 ± 54.5
TD‐5	581 ± 49.5
TD‐6	574 ± 47.4
TD‐7	593 ± 47.7
TD‐8	609 ± 50.2
TD‐9	616 ± 50.6
TD‐10	634 ± 50.2
TD‐11	664 ± 54.0
TD‐12	700 ± 56.8
TH (pitch = 0.397)	712 ± 78.8
TH (pitch = 0.430)	679 ± 72.9

TD‐n: TomoDirect n‐port (*n* = 2–12), TH: TomoHelical.

TD‐2 plan has AP/PA beam configuration.

## 4 Discussion

The number of ports used in TomoDirect planning can significantly influence dose distribution and beam‐on time. This study evaluated the impact of the number of ports on lower body TBI using TomoDirect and compared the results with those of TomoHelical methods. Dose uniformity was superior with a TomoHelical pitch of 0.397 compared to 0.430, and was further improved with TomoDirect, except for the five‐port configuration. Figure 2 illustrates the dose distributions of the TomoHelical and two‐port TomoDirect plans for the same patient. A ripple‐like dose variation pattern, referred to as the thread effect,^18^, ^19^ was observed in the TomoHelical plans, which was more pronounced at the 0.430 pitch. In contrast, the TomoDirect plan did not exhibit such variation. Similar findings in upper‐body TBI have been reported by Kasai et al.^8^

The thread effect becomes more pronounced with increasing off‐axis distance but can be mitigated by selecting an optimal pitch value. Kissick et al. reported that pitch values determined by 0.86/n (n: integer) minimize the thread effect at an off‐axis distance of 5 cm.^18^ Chen et al. further analyzed the thread effect theoretically and identified 0.397 as the optimal pitch to minimize dose ripples at an off‐axis distance of 20 cm with an FW of 5.0 cm.^19^ As shown in Figure 2, in the lower‐body region extending from the feet to the mid‐thigh—where the target volume lies primarily beyond 5 cm off‐axis—the 0.397 pitch effectively suppressed the thread effect. Nevertheless, in TomoHelical delivery, residual helical‐specific dose modulation can still produce periodic dose fluctuations along the longitudinal direction, resulting in localized low‐dose regions (cold spots) within the target. To maintain adequate target coverage and ensure that these cold spots satisfy the prescribed minimum dose, the inverse optimization algorithm compensates by increasing the intensity of neighboring beamlets. This compensation inevitably elevates the maximum dose within the target volume, leading to higher D2 values in TomoHelical compared with TomoDirect. Consequently, because the HI reflects the spread between high‐ and low‐dose regions, the greater dose variability associated with TomoHelical resulted in significantly poorer HI values. In contrast, no ripple artifacts were observed in the TomoDirect plans. This absence is attributable to the lack of helical beam junctioning in TomoDirect mode, which is the primary cause of the thread effect in TomoHelical. Consequently, TomoDirect provides a more homogeneous dose distribution than TomoHelical.

**FIGURE 4:**
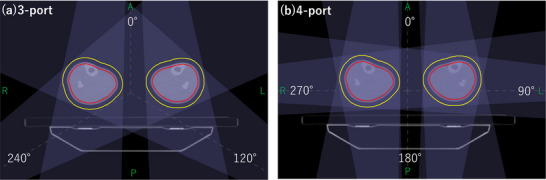
Examples of the geometric relationship between the lower‐body target and beam angles in TomoDirect for patient 12, whose body mass index corresponds to the median of the cohort. (a) TomoDirect plan with three‐port, (b) TomoDirect plan with four‐port. The red and yellow lines represent PTV1 and PTV2, respectively. In the three‐port configuration, differences in beam intensity across the target associated with the flattening‐filter‐free beam are pronounced at 120° and 240°. In the four‐port configuration, pronounced effects of beam attenuation caused by the traversal of the proximal target before reaching the contralateral target, as well as air gap effects, arise at 90° and 270°. In contrast, in both plans, the beams at 0° and 180° are minimally affected by these effects.

According to a previous study, TomoDirect plans for upper‐body TBI tend to improve in all dose evaluation indices as the number of ports increases.^8^ However, for lower‐body irradiation, no such improvement was observed when the number of ports increased from three to five. This discrepancy may be explained by anatomical and geometric differences: in lower‐body TBI, the target consists of two parallel cylindrical structures corresponding to the bilateral legs, separated by an air gap (Figure 4). At certain beam angles, the beam traverses proximal target volume before reaching the contralateral target, causing attenuation. Moreover, an air gap along the beam path gives rise to build‐up and build‐down effects at air–tissue interfaces. These effects are absent in the more contiguous, approximately cylindrical target geometry of the upper body. Furthermore, the TomoTherapy system employs a flattening‐filter‐free (FFF) beam with a conical intensity profile, and some oblique beam angles may therefore contribute less effectively to dose homogeneity depending on their spatial relationship to the target. When only a limited number of ports are employed, the angular coverage is insufficient to compensate for dose inhomogeneities introduced by these geometric and dosimetric factors. In this study, the MF was set to 1.5, limiting the degree of leaf modulation available to compensate for beam attenuation caused by the traversal of the proximal target before reaching the contralateral target and air gap effect related dose perturbations using FFF beam. By contrast, Kasai et al. used a higher MF in upper‐body TBI, allowing greater benefit from increased beam angles.^8^ Within the range of three to five ports, although increasing the number of ports can provide angular compensation, the combined effects of target separation, FFF beam characteristics, and restricted modulation outweighed this benefit. Consequently, the trend of improved dose homogeneity reported for upper‐body TBI was not observed.^8^

In the two‐port TomoDirect plans, significant differences in all dose evaluation indices were observed between the AP/PA and LR/RL beam configurations. In particular, D2 and HI were notably worse with the LR/RL configuration, indicating that beam configuration plays a critical role in dose distribution. Optimizing beam angles may therefore yield improved outcomes, regardless of the number of ports used. Further investigation is warranted to clarify the influence of beam configuration in greater detail.

The two‐port TomoDirect plan with AP/PA configuration demonstrated superior dose evaluation indices and the shortest beam‐on time among all compared plans. Regarding dose homogeneity, the two‐port AP/PA plan outperformed the TomoHelical plans and was comparable to TomoDirect plans with eight or more ports. On average, the beam‐on time for the two‐port AP/PA plan was more than 120 s shorter than that of both the TomoHelical plan with a 0.397 pitch and the TomoDirect plan using a maximum of twelve ports. This marked reduction in beam‐on time may improve patient comfort during treatment and should be considered a safety benefit, as shorter irradiation times reduce the risk of intra‐fraction motion, which is particularly relevant for patients undergoing TBI, who may experience pain or discomfort during prolonged irradiation, including pediatric and frail patients who are more vulnerable to prolonged immobilization. However, in clinical practice, the total treatment time for TomoDirect mode also includes couch repositioning time. The two‐port AP/PA configuration reduces the frequency of couch movements, thereby minimizing the overall treatment duration. Based on these results, the two‐port TomoDirect plan with an AP/PA beam configuration appears to be a clinically practical and efficient approach for lower body TBI. These findings may inform institutional TBI protocols by supporting the use of a simplified two‐port AP/PA TomoDirect strategy as a standard option for lower‐body irradiation, particularly in clinical settings where treatment efficiency and patient comfort are prioritized. Furthermore, TomoTherapy's extensive longitudinal coverage eliminates target segmentation required by conventional C‐arm or other O‐ring linear accelerator, resulting in a more efficient delivery and simplified the treatment planning workflow.

This study has several limitations. First, robustness analyses, including setup uncertainty and junction sensitivity, were not performed. In particular, dose inhomogeneities may arise at the junction region located at the mid‐thigh due to setup errors, and additional strategies such as gradient matching required to mitigate these effects.^4^ Second, this study was based solely on treatment planning system simulations, and no experimental verification such as phantom measurements or correlation with clinical outcomes was conducted. Therefore, further evaluation, including robustness assessment focusing on junction management strategies and experimental validation for the two‐port TomoDirect plan with an AP/PA configuration, is necessary to ensure safe clinical implementation and to establish more robust and validated protocols.

## 5 Conclusions

We investigated the dosimetric impact of the number of ports on the dose evaluation indices for lower body TBI using the TomoDirect mode, including comparisons with TomoHelical. TomoDirect generally provides more homogeneous dose distributions than TomoHelical, except when five ports are used. Notably, the two‐port TomoDirect plan with the AP/PA beam configuration achieved dose homogeneity comparable to that of plans using eight or more ports. Considering both dose homogeneity and beam‐on time, the two‐port TomoDirect plan with AP/PA configuration may be considered a clinically reasonable and efficient option for lower body irradiation, offering the additional clinical advantage of reducing the risk of intra‐fraction motion shorter treatment times. Future studies incorporating robustness analyses and experimental validation will be essential to validate the proposed approach and to facilitate its safe integration into clinical practice.

## AUTHOR CONTRIBUTIONS

Conception and design: Yoshiki Suetsugu, Yuka Yamanaka, Keiki Inoue, Tomonari Sasaki. Administrative support: Tomonari Sasaki. Provision of study materials or patients: Naoki Higashi, Yukihide Fukuyama, Hiromi Terashima. Data analysis and interpretation: Yoshiki Suetsugu, Naoki Higashi, Yukihide Fukuyama, Shun Nakano. Manuscript writing: Yoshiki Suetsugu, Tomonari Sasaki. Final approval of manuscript: Yoshiki Suetsugu, Naoki Higashi, Yukihide Fukuyama, Yuka Yamanaka, Keiki Inoue, Shun Nakano, Hiromi Terashima, Tomonari Sasaki.

## CONFLICT OF INTEREST STATEMENT

The authors declare no conflicts of interest.

## Data Availability

All data generated or analyzed during this study are included in this published article.
